# Supplementary material for: Analysis of PPARγ Signaling Activity in Psoriasis
Source: Int J Mol Sci. 2021 Aug 10;22(16):8603. doi: 10.3390/ijms22168603 (PMC8395241; doi:10.3390/ijms22168603)
Supplement: Supplementary file 1 [file ijms-22-08603-s001.zip › Supplemental materials_Analysis of PPARg signaling activity in psoriasis/Pathway models/Models images and html files/Interleukin-17 and interleukin-22 signaling in psoriasis/3649.html]

DSC1


# Protein DSC1

|  |  |
| --- | --- |
| URN | urn:agi-llid:1823 |
| Total Entities | 0 |
| Connectivity | 161 |
| Name | DSC1 |
| Description | desmocollin 1 |
| Notes | The protein encoded by this gene is a calcium-dependent glycoprotein that is a member of the desmocollin subfamily of the cadherin superfamily. These desmosomal family members, along with the desmogleins, are found primarily in epithelial cells where they constitute the adhesive proteins of the desmosome cell-cell junction and are required for cell adhesion and desmosome formation. A subtype of IgA pemphigus, a life-threatening autoimmune disease, is characterized by the presence of autoantibodies that target the encoded protein. The desmosomal family members are arranged in two clusters on chromosome 18. Alternative splicing of this gene results in multiple transcript variants. At least one of these variants encodes a preproprotein that is proteolytically processed to generate the mature protein. [provided by RefSeq, Nov 2015] |
| Primary Cell Localization | Plasma membrane |

---

|  |  |
| --- | --- |
| Pathway | Desmosome Assembly |
|  | Desmosome Dysfunction in Cardiomyocyte |
|  | Desmosomes Role in Dilated Cardiomyopathy |
|  | Corneodesmosomes in Atopic Dermatitis |
|  | Proteins with Altered Expression in Atopic Dermatitis |
|  | Atopic Dermatitis |
|  | IL22 Induces Keratinocyte Proliferation in Psoriasis |
|  | 2\_Interleukin-17 and interleukin-22 signaling in psoriasis |
|  | 2\_Harlequin ichthyosis overview |
|  | 2\_Desmosome dysfunction in cardiomyocytes in ARVD |
|  | Interleukin-17 and interleukin-22 signaling in psoriasis |

---

|  |  |
| --- | --- |
| MedScan ID | 1823 |

---

|  |  |
| --- | --- |
| LocusLink ID | 1823 |
|  | 13505 |
|  | 291759 |

---

|  |  |
| --- | --- |
| Alias | CDHF1 |
|  | desmocollin 1 |
|  | Desmocollin 1A/1B |
|  | AI507491 |
|  | DSC1 |
|  | Dsc1b |
|  | desmocollin I |
|  | isoform Dsc1b preproproteins |
|  | RIKEN cDNA 1110020A10 gene |
|  | Dsc1a |
|  | isoform Dsc1a preproproteins |
|  | Desmosomal glycoprotein 2/3 |
|  | 1110020A10 |
|  | DSC I |
|  | MGC129348 |
|  | 1110020A10Rik |
|  | CDHF I |
|  | DG2/DG3 |
|  | cadherin family member 1 |
|  | desmocollin-1 |

---

|  |  |
| --- | --- |
| GO ID | 0005509 |
|  | 0098609 |
|  | 0070268 |
|  | 0007156 |
|  | 0031424 |
|  | 0043312 |
|  | 0005911 |
|  | 0001533 |
|  | 0030057 |
|  | 0070062 |
|  | 0101003 |
|  | 0005921 |
|  | 0016021 |
|  | 0016020 |
|  | 0005886 |
|  | 0007155 |
|  | 0030054 |

---

|  |  |
| --- | --- |
| KEGG ID | hsa:1823 |
|  | mmu:13505 |

---

|  |  |
| --- | --- |
| Organism | Homo sapiens {Organism urn:agi-taxid:9606} |
|  | Mus musculus {Organism urn:agi-taxid:10090} |
|  | Rattus norvegicus {Organism urn:agi-taxid:10116} |
|  | Homo sapiens |
|  | Mus musculus |
|  | Rattus norvegicus |

---

|  |  |
| --- | --- |
| Mouse chromosome position | 18 |

---

|  |  |
| --- | --- |
| OMIM ID | 125643 |

---

|  |  |
| --- | --- |
| Rat chromosome position | 18p12 |

---

|  |  |
| --- | --- |
| Hugo ID | 3035 |

---

|  |  |
| --- | --- |
| Human chromosome position | 18q12.1 |

---

|  |  |
| --- | --- |
| Swiss-Prot Accession | Q08554 |
|  | Q9HB00 |
|  | Q08554.2 |
|  | P55849 |
|  | Q2VPA9 |
|  | Q32ME9 |
|  | P55849.2 |
|  | Q9HB01 |
|  | E9QJX2 |

---

|  |  |
| --- | --- |
| PIR ID | A48910 |
|  | I37281 |
|  | I37282 |

---

|  |  |
| --- | --- |
| GenBank ID | NC\_000018 |
|  | NM\_024421 |
|  | NP\_077739 |
|  | NM\_004948 |
|  | NP\_004939 |
|  | NG\_029192 |
|  | AC012417 |
|  | AF293358 |
|  | AAG23424 |
|  | AAG23425 |
|  | CH471088 |
|  | EAX01252 |
|  | EAX01253 |
|  | Y11051 |
|  | CU444724 |
|  | CU447407 |
|  | X72925 |
|  | CAA51428 |
|  | CAA51429 |
|  | Z34522 |
|  | CAA84278 |
|  | CAA84279 |
|  | Q08554 |
|  | NC\_000084 |
|  | NM\_001291804 |
|  | NP\_001278733 |
|  | NM\_013504 |
|  | NP\_038532 |
|  | AC132314 |
|  | AF069759 |
|  | AAD04343 |
|  | CH466557 |
|  | EDK96941 |
|  | EDK96942 |
|  | AI507491 |
|  | AK003840 |
|  | AK036893 |
|  | AK079441 |
|  | AK081826 |
|  | BAC38343 |
|  | BC109161 |
|  | AAI09162 |
|  | BC109162 |
|  | AAI09163 |
|  | X97986 |
|  | CAA66628 |
|  | CAA66629 |
|  | P55849 |
|  | NC\_005117 |
|  | XM\_008771992 |
|  | XP\_008770214 |
|  | AC\_000086 |
|  | XM\_017587802 |
|  | XP\_017443291 |
|  | AABR07031382 |
|  | AABR07031383 |
|  | AAHX01093939 |
|  | AAHX01093940 |
|  | AAHX01093941 |
|  | CH473974 |
|  | EDL76086 |
|  | DN948066 |
|  | NC\_018929 |
|  | AMYH02034503 |
|  | AMYH02034504 |
|  | BC148349 |
|  | AAI48350 |
|  | AC\_000040 |
|  | AAHY01136938 |
|  | AAHY01136939 |
|  | XM\_008774118 |
|  | XP\_008772340 |

---

|  |  |
| --- | --- |
| Swiss-Prot ID | DSC1\_HUMAN |
|  | DSC1\_MOUSE |

---

|  |  |
| --- | --- |
| Cell Localization | Cell membrane |
|  | Cell junction |
|  | Desmosome |
|  | Cell junction, desmosome |

---

|  |  |
| --- | --- |
| Ensembl ID | ENSG00000134765 |
|  | ENSP00000257197.3 |
|  | ENST00000257197.7 |
|  | ENSP00000257198.5 |
|  | ENST00000257198.5 |
|  | ENSMUSG00000044322 |
|  | ENSMUSP00000153639.1 |
|  | ENSMUST00000224432.1 |
|  | ENSMUSP00000042303.5 |
|  | ENSMUST00000038710.5 |
|  | ENSRNOG00000056258 |
|  | ENSRNOP00000075307.1 |
|  | ENSRNOT00000078207.1 |
|  | ENSP00000257197 |
|  | ENST00000257197 |
|  | ENSP00000257198 |
|  | ENST00000257198 |
|  | ENSMUSP00000042303 |
|  | ENSMUST00000038710 |
|  | ENSRNOP00000075307 |
|  | ENSRNOT00000078207 |

---

|  |  |
| --- | --- |
| MGI ID | MGI:109173 |

---

|  |  |
| --- | --- |
| RGD ID | 1310768 |

---

|  |  |
| --- | --- |
| Unigene ID | Hs.567260 |
|  | Mm.33740 |
|  | Mm.482847 |

---

|  |  |
| --- | --- |
| Homologene ID | 22761 |

---
